# Active KTaO_3_ hybrid terahertz metamaterial

**DOI:** 10.1038/s41598-017-05529-0

**Published:** 2017-07-20

**Authors:** Liang Wu, Jinglong Liu, Hui Li, Chunfeng Ding, Ningning Xu, Xiaolei Zhao, Zongcheng Xu, Quan Sheng, Jianquan Yao, Jining Li, Xin Ding, Weili Zhang

**Affiliations:** 1 College of Precision Instrument and Optoelectronics Engineering, Tianjin University, Key Laboratory of Optoelectronics Information and Technology (Ministry of Education), Tianjin, 300072 China; 2 School of Electrical and Computer Engineering, Oklahoma State University, Stillwater, Oklahoma 74078 USA; 3 College of Physics, Jilin University, Changchun, Jilin 130012 China; 4 Henan Key Laboratory of Laser and Opto-electric Information Technology, Zhengzhou University, Zhengzhou, 450052 China

## Abstract

The dielectric properties of an active KTaO_3_ hybrid metamaterial structure and its tunability under external electric fields are investigated at room temperature by means of terahertz time-domain spectroscopy. Application of the electric field leads to an appreciable tuning of the dielectric loss, which is up to 17%. Meanwhile, the refractive index also changes appreciably. These findings are attributed to the internal space charge field in the crystal caused by the excited free carriers.

## Introduction

The terahertz frequency range, overlapping with many fundamental elementary excitations of physical systems such as molecular vibrations, phonons, quasi-free electrons, excitons, and magnons, has become extensively exploited during the past two decades^1, 2^. In order to achieve active control of the terahertz wave propagation, it is an important issue to investigate tuning properties of various materials and structures in the terahertz regime. Metamaterials, offering unprecedented functionalities to manipulate electromagnetic waves, have become a research hotspot in recent years. Through incorporation of active media, the exotic electromagnetic behavior of metamaterials could be dramatically empowered by dynamic control^3–5^. Ranjan Singh *et al*. reported complex oxide based tunable active terahertz metamaterials for different applications such as photoswitching^6–10^.

Potassium tantalate (KTaO_3_, KTO), like Strontium titanate (SrTiO_3_, STO), is one of the most popular incipient ferroelectrics with interesting potential for technical application, in which the quantum fluctuations at low temperatures prevent a displacive ferroelectric phase transition to occur. Recently, research on properties of KTO in the terahertz spectral range has drawn the attention of many researchers^11–17^. V. Skoromets *et al*. prepared a polycrystalline TO thin film by using a chemical solution deposition method, and measured its permittivity at terahertz frequencies at different temperatures^11^. Sebastjan Glinsĕk *et al*. reported the broad-band dielectric properties and lattice dynamics of KTO ceramics, revealing that the soft mode frequency agrees well with the values that in single crystal^12^. We have investigated the dielectric property of KTO single crystals in the terahertz range, and found that the dielectric constant can be modulated by an optical field at room temperature, which decreased ~3.5% with light excitation power of 600 mW^13^.

Thus, the dielectric properties in bulk KTO under electrical field obtained by terahertz spectroscopy have rarely been explored, and only a few observations of the tunability of KTO single crystal at room temperature has been reported up to now. V Skoromets *et al*. studied the electric-field tunability of the dielectric properties of KTO single crystal by using terahertz spectroscopy in a broad temperature range^18^, the relative change of the real part of the permittity could reach up to ~12% at 60 K upon the electric field of 50 kV cm^−1^. Moreover, additional effects could also be observed in the crystal with designed structures, which cannot be described by the conventional model. P. Kužel *et al*. have developed an approach with an interdigited electrode structure to be used to determine the electric field dependence of the complex permittivity of a STO thin film on a sapphire substrate^19^. With a 100 kV/cm electric field applied, they demonstrated up to 10% variation of the film permittivity at 300 GHz at room temperature. In this letter, we present a KTO based hybrid metamaterial structure and the tunability of its dielectric properties under external electrical excitation. We observe that a low DC bias would change both the refractive index and absorption coefficient, consequently modulate the terahertz response in the hybrid structure. Variation of absorption coefficient at the peak could reach up to 17%. The proposed KTO metamaterial offers a reference for developing high-performance real world photonic devices for terahertz technology.

## Methods

**Figure 1:**
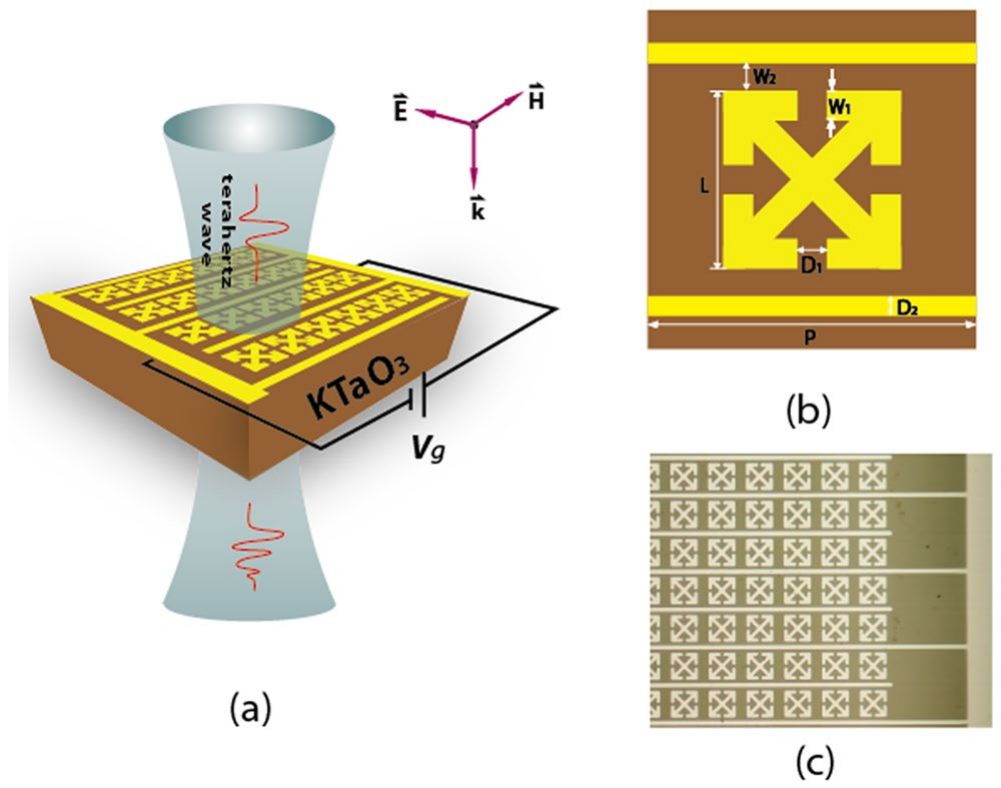
(**a**) Experimental design and illustration of the sample. The KTO crystal was biased with voltage *V*_*g*_. (**b**) Schematic of the unit structure with parameters: P = 50 μm, L = 35 μm, W_1_ = D_1_ = 4 μm, W_2_ = 2.5 μm, D_2_ = 5 μm. (**c**) Microscopic image of the fabricated KTO hybrid metamaterial.

The KTO crystals (MTI Corporation) are 10 mm × 10 mm × 0.535 mm in dimensions with an orientation of <100>. The periodic interdigitated electrode metastructure is patterned using conventional photolithography on the KTO bulk crystal, followed by 180-nm Aluminum film deposition. We used a photoconductive switch based 8 f terahertz time-domain spectrometer (THz-TDS) to measure the transmittance spectra through the KTO hybrid structure, as shown in Fig. 1. The samples are excited by a 3.5-mm beam waist at normal incidence with a sample area of 1 cm^2^. The detail schematic of the unit metastructure of with parameters is shown in Fig. 1(b). With the electric field perpendicular to the interdigitated electrode, the transmission in time and frequency domain are measured for the sample and reference. A free space signal is used as the reference. By applying a constant electrical voltage, we discovered that the transmitted terahertz signals could be dynamically modulated. All measurements were carried out at room temperature in a dry air environment in order to eliminate the absorption of terahertz waves by water vapor present in the atmosphere. No arcing is observed during the measurement. The electrode separation is 40 μm, so the maximum electric field intensity is less than 30 kV/cm.

## Results and Discussion

**Figure 2:**
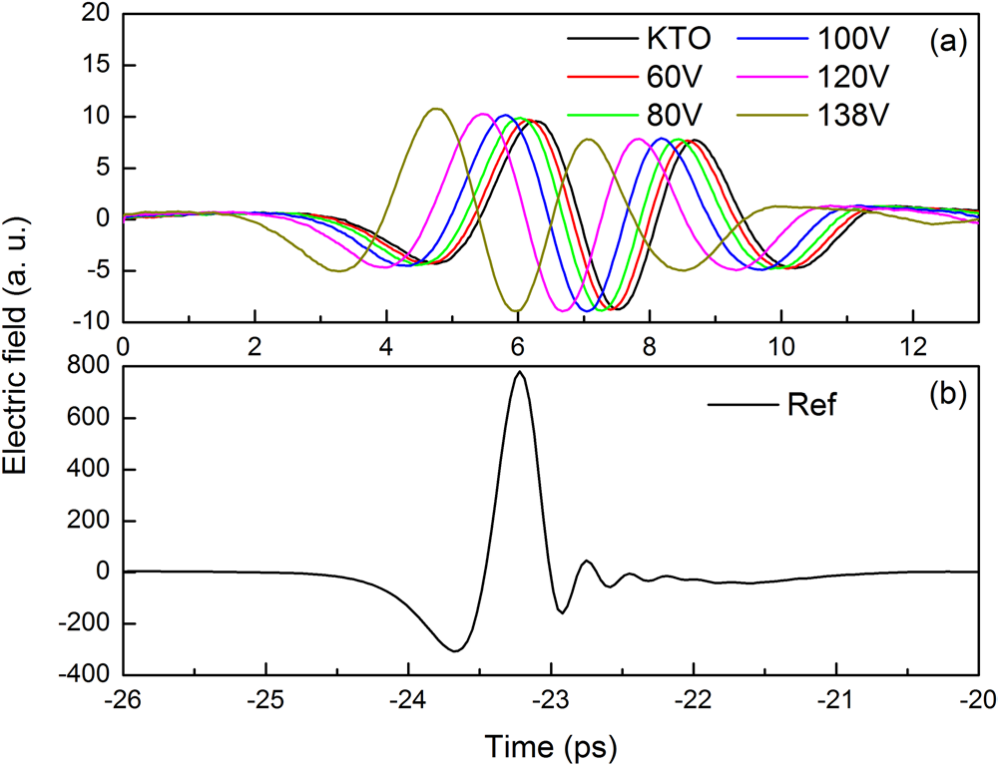
(**a**) Time domain transmittance waveform of KTO hybrid metamaterial sample and its time shift under external voltages at room temperature. (**b**) The reference waveform is also plotted.

Figure 2 shows typical experimental results of the terahertz time domain waveforms passing through the sample. The time lag between the reference and sample is around 30 ps, as seen in Fig. 1. The signal waveforms transmitted through the KTO hybrid metamaterials show appreciable change with the increase of bias voltage. For example, when the applied bias is 138 V, the transmittance waveform shifts about 1.5 ps and the amplitude increases from 9.5 to 10.8, compared to that without electric excitation.

**Figure 3:**
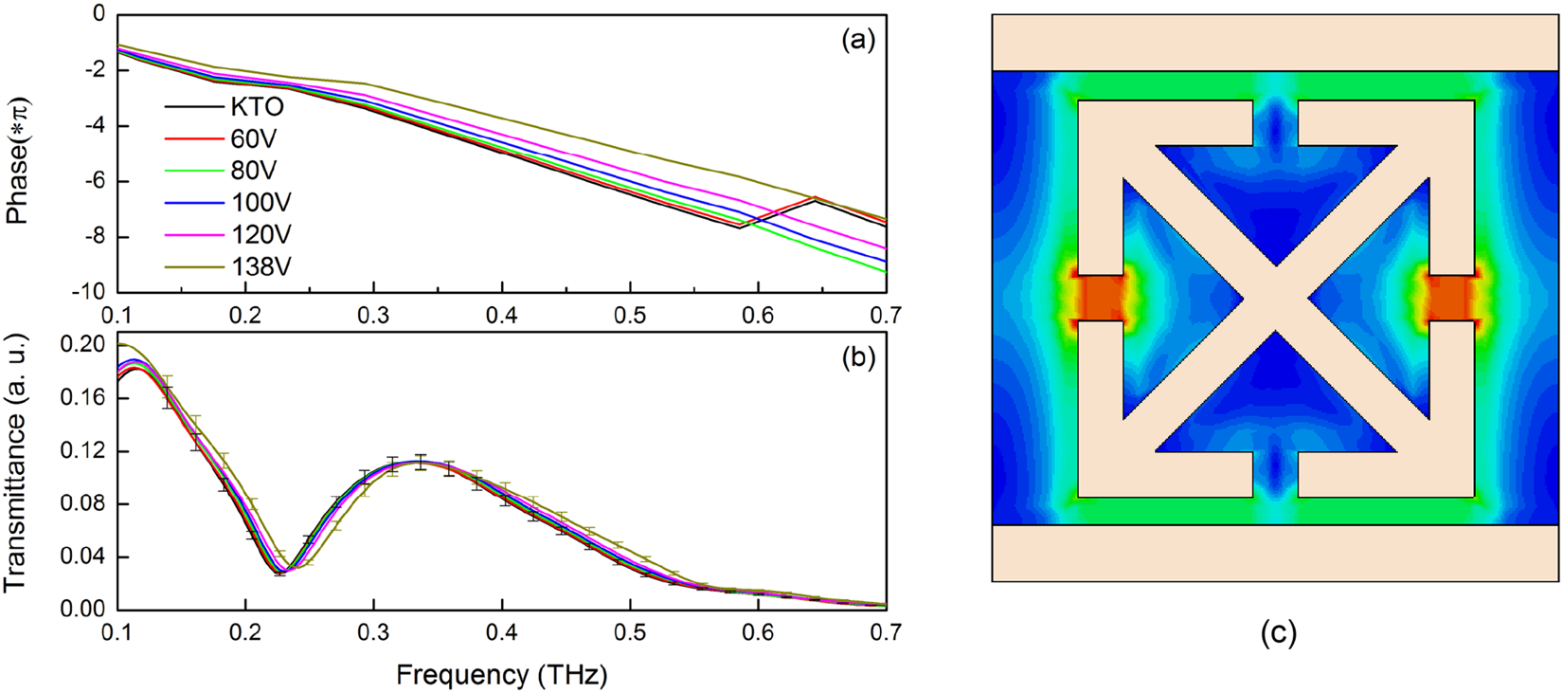
(**a**) Phase change and (**b**) transmission spectrum of KTO hybrid metamaterial sample under external voltages at room temperature. (**c**) Simulated electric field distribution of the LC resonator structure at resonance frequency.

Through a Fourier transform, we calculated the complex transmittances and the intrinsic phases in frequency domain from the waveforms in Fig. 2. Figure 3 shows examples of terahertz complex dielectric spectra of the samples under different external electric fields. The detectable frequency range was from 0.1 to 0.7 THz (3.3 cm^−1^ to 23.1 cm^−1^), since the KTO samples are nearly opaque above ~1 THz. At zero gate current, a pronounced LC resonance approximately at 0.23 THz could be observed, which is determined by *f*_0_ = *ω*/*2π* = 1/*2π* (*LC*)^1/2^ ^20^. Here *L* is the inductance of the square loop and *C* is the capacitance of the gap. Both phase and transmittance amplitude increase with an increasing pump voltage, namely, the corresponding refractive index and dielectric loss decrease with the voltage growing.

Figures 2 and 3(a) demonstrate that the phase and time position of the transmittance signals also show differences, indicating that the refractive index changes considerably (5~8%) with external electric field. Compared to our previous work^13^, the new structure, delivering a modulation depth of more than 17% for absorption loss, greatly enhanced the dielectric tunability of the KTO crystal. Figure 3(c) shows simulated electric field distribution of the LC resonator structure at resonance frequency using CST Microwave studio frequency-domain solver. The LC resonator design supports strongly confined electric field in the capacitive gap at fundamental inductive-capacitive resonance mode.

**Figure 4:**
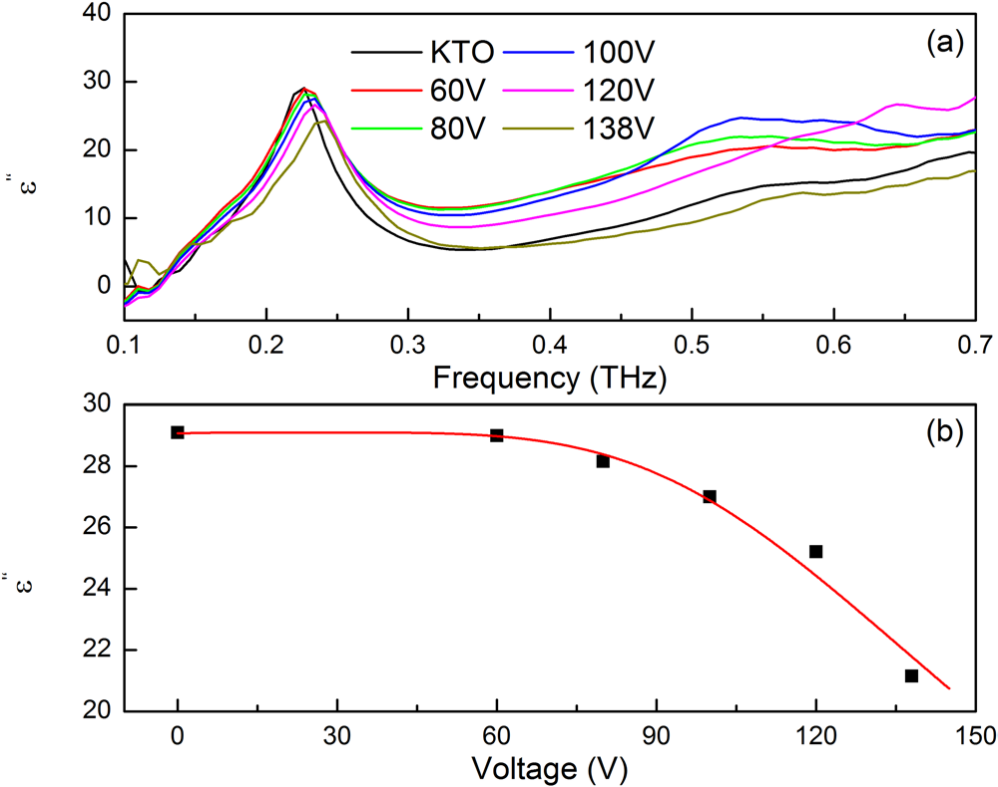
(**a**) Imaginary part of permittivity of KTO hybrid metamaterial sample and its variation under external voltages at room temperature. (**b**) Variation of *ε”* at the peak.

In order to illustrate the bias induced ferroelectric properties in KTO, it is worthwhile to study the variation of its dielectric loss under the appearance of external electric fields. The thickness of the metal structure is 180 nm, which is much smaller than that of the KTO crystal (0.535 mm). For simplicity, we considered the LC resonator-KTO two-layer structure as a uniform effective dielectric media, the imaginary part of permittivity (dielectric loss) was then retrieved from the terahertz transmittance spectra. Figure 4(a) shows the imaginary part of permittivity of the KTO hybrid metamaterial and its variation under external voltages at room temperature. Figure 4(b) demonstrates modulation of the imaginary part of permittivity as a function of the applied electric power and its fit. In the frequency range of 0.1–0.7 THz, the active KTaO_3_ hybrid metamaterial structure does not show a giant dielectric loss, the magnitude of *ε”* is about 3–15, nearly the same with that of KTO crystal without any structure^13^. We notice that the peak positon of *ε”* shifts obviously with different levels of voltage bias, indicative of the tuning of LC resonance frequency. The maximum frequency shift could reach up to 0.02 THz when the applied voltage is up to 138 V. The measured dielectric loss is found to be tunable by up to 17% via the application of an external electric field, and the dielectric constant nonlinearly decreases with increaseing voltage bias. The measured dielectric loss of the hybrid structure versus applied voltage at the peak (corresponding to the resonance frequency) are shown in Fig. 4, which fits well with the following empirical expression^21^:

1 $$\frac{{\varepsilon }^{{\rm{^{\prime} }}{\rm{^{\prime} }}}(V)}{\varepsilon {{}^{{\rm{^{\prime} }}{\rm{^{\prime} }}}}_{0}}=\frac{1}{{(1+{\alpha }^{3}{V}^{2})}^{2/3}}$$

where *ε”*_0_ and *ε”*(*V*) are the imaginary part of dielectric constants under zero electric field and under the applied bias, respectively, and *α* is the anharmonic coefficient. The anharmonic coefficient is assumed to be an order parameter of the anharmonic interactions, and we can fit the profile well using α = 8.476 × 10^−9^ at the peak of *ε”*. The fitting results using equation (1) are shown as solid lines in Fig. 4(b).

It is noticed that with only parallel electrodes, a broadband tuning effect for the sample could be achieved, but we still believe that the resonators are necessary. Firstly, the active LC metasurface can be generalized to function at defined resonance frequencies. In the measurement, the resonance frequency shifted about 0.02 THz with the applied bias, which may be used for phase tuning. Secondly, the LC resonator design strongly supports confined electric field in the capacitive gap at fundamental LC resonance mode, indicating that the tuning effect could be enhanced more or less at resonance frequency (maybe not obvious).

The interesting characteristics observed in the active modulation of the absorption coefficient in the metamaterial are mainly attributed to the modification by the free carriers in the hybrid structure with varying gate voltages. Usually there are only Ta^5+^ ions in the KTO single crystals; the local defects could also result in the presence of Ta^4+^ ions in the samples^22, 23^. We neglect thermal excitations here. When a voltage bias is applied to the sample, a high electric field intensity for the KTO crystal could be acquired, and a lot of free electrons would be generated because there is an exchange of electrons between Ta^4+^ and Ta^5+^ ions. The electrons in Ta^4+^ ions can hop to the conduction band, they migrate in a preferred direction, and finally are captured by the trap level of Ta^5+^. The dominant charge driving force during this process is supposed to be the voltaic effect. Drift and diffusion could also have contribution to the movement of the electrons. The migration process would lead to a redistribution of carriers in the KTO crystal, then the space displacement of carriers leads to an internal space charge field that shields the external electric field until a new equilibrium is reached. This internal space charge field changes the dielectric properties of the sample through electro-optic effect^21, 24^, as shown in Fig. 4.

We should also clarify that in the process, the quantity of free carriers does not change, so the tuning of complex dielectric constant can be attributed to the electro-optic effect. If the tuning effect mainly comes from the carriers, the absorption coefficient would be tuned significantly, but the real part of permittivity will not show obvious change. In this measurement, both the real and imaginary parts of the dielectric constant change simultaneously with the same trend with electric fields, so we believe that the electro-optic effect plays a dominant role in the tuning effect. Some of our previous work also supported this conclusion^25^.

### Summary

We investigated the frequency dependence of dielectric response of a new LC resonator-KTO metamaterial and its electric-field-induced tunability by using THz-TDS at room temperature. An appreciable variation of absorption coefficient in KTO crystal was also demonstrated with different levels of external electric fields. This property originates from the voltaic effect caused by the electric-induced free carriers as well as their shielding effect, leading to an internal space charge field. It is supposed that the two mechanisms complement with each other, resulting in the observed dielectric tuning behaviors of KTO. Such a variation in the dielectric response could be used in phase or amplitude modulator in the terahertz regime. The findings would illuminate further applications for novel functional integrated devices based on KTO or other ferroelectric materials.
